# 315. Modeling Strain-Dependent Variability in Carbapenem-Resistant *Klebsiella pneumoniae* Colonization in the Malnourished Host

**DOI:** 10.1093/ofid/ofae631.105

**Published:** 2025-01-29

**Authors:** Thomas Holowka, Jamie Xiao, Kalisvar Marimuthu, Oon Tek Ng, David van Duin, Luther A Bartelt

**Affiliations:** University of North Carolina, Chapel Hill, Chapel Hill, NC; University of North Carolina, Chapel Hill, North Carolina; National Centre for Infectious Diseases, Singapore, Not Applicable, Singapore; National Centre for Infectious Diseases, Singapore, Not Applicable, Singapore; University of North Carolina at Chapel Hill, Chapel Hill, NC; University of North Carolina School of Medicine, Chapel Hill, NC

## Abstract

**Background:**

Particular strains of Carbapenem-resistant *Klebsiella pneumoniae* (CR-Kp) have achieved global dominance extending to developing countries where malnutrition is a potential risk factor for infection. Bacteria and host properties that determine colonization dynamics in this context are not understood. We hypothesized that strain variability in host cell adhesion and nutrient-utilization *in vitro* predicts *in vivo* intestinal colonization by CR-Kp in malnourished hosts.

**Figure 1:**
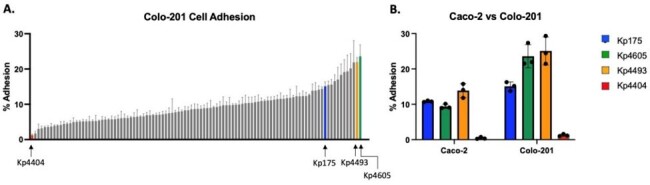
Strain-dependent cell adhesion by carbapenem-resistant Klebsiella pneumoniae (A) Adhesion to Colo-201 cells and/or (B) Caco-2 cells as percentage of bacteria recovered after co-culturing then washing off non-adherent cells. Bacterial quantification performed by culturing on MacConkey agar + Meropenem. Strains of interest indicated with arrows and colored-bars. All experiments showing results of 3 replicates.

**Methods:**

Patient-derived isolates of CR-Kp were subject to *in vitro* assays testing adhesion to human colon cell lines. Select isolates were further tested for *in vitro* growth in minimal media with limiting protein availability. These isolates were additionally inoculated via orogastric lavage into C57BL/6 mice (n=6 per group) on a nutrient replete control diet (CD) or isocaloric protein-deficient diet (PD) and intestinal colonization was quantified with selective culture of feces and intestinal specimens on MacConkey agar containing meropenem.

**Figure 2:**
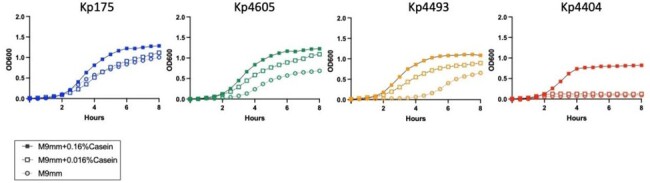
Nutrient limited growth of carbapenem-resistant Klebsiella pneumoniae Growth in vitro determined by serial absorption measurement of bacteria culture under different nutrient conditions. M9 minimal medium (M9mm; M9 solution +0.4% glucose) was used with or without casein peptone (0.16% or 0.016%) as a protein/amino acid source.

**Results:**

Across >100 screened human isolates of CR-Kp, adhesion to human colon cell lines ranged from < 1% to 30% (Figure 1). Three high-adhesion isolates (Kp175, Kp4493 and Kp4605) and one low-adhesion isolate (Kp4404) were selected for further investigation (Table 1). All three high-adhesion isolates grew to varying degrees in protein-limited conditions, while Kp4404 required a protein source for growth (Figure 2). Mice inoculated with CR-Kp were stably colonized only if they were fed a PD rather than CD diet. Kp175 and Kp4605 demonstrated the highest fecal shedding and intestinal burden (10^2^-10^4^ CFU per mg), which was significantly greater on a PD versus CD diet (p< 0.05 by Mann-Whitney test). In contrast, Kp4493 and Kp4404 inconsistently colonized mice regardless of diet (Figure 3).

**Figure 3:**
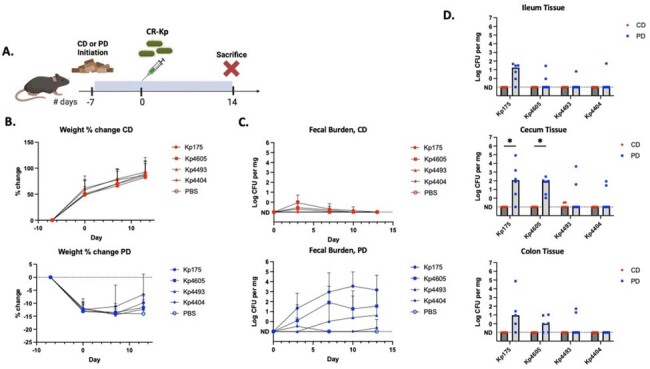
Diet-dependent intestinal colonization by carbapenem-resistant Klebsiella pneumoniae (A) 3-4 wk old C57BL/6 male mice initiated on nutrient-replete control diet (CD) or isocaloric protein-deficient diet (PD) and inoculated via orogastric lavage with 10^6 CFU of carbapenem-resistant Klebsiella pneumoniae (CR-Kp). (B) Weight measured over time along with bacterial burden in (C) fecal pellets and (D) intestinal tissue by culturing on MacConkey agar + Meropenem. 6 animals used per condition. * = p<0.05 by Mann Whitney Test.

**Conclusion:**

Our findings suggest a wide variability in intestinal cell adhesion and nutrient utilization by clinical isolates of CR-Kp. These *in vitro* properties incompletely predict *in vivo* colonization in a mouse model. Notably, host dietary deficiency promotes intestinal colonization by certain nutritionally resilient CR-Kp isolates, with potential implications for the global spread of high-risk strains in malnourished populations.

**Table 1:**
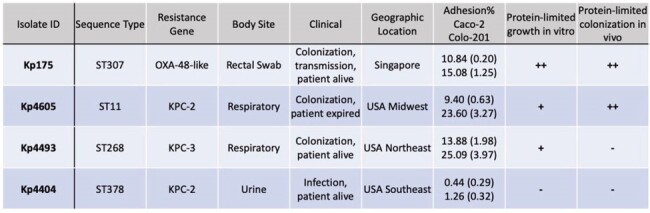
Genotypic, clinical and phenotypic characteristics of carbapenem-resistant Klebsiella pneumoniae

Adhesion percentage express as mean (standard deviation).

**Disclosures:**

**David van Duin, MD, PhD**, Merck: Advisor/Consultant|Merck: Grant/Research Support|Pfizer: Advisor/Consultant|Qpex: Advisor/Consultant|Roche: Advisor/Consultant|Shionogi: Advisor/Consultant|Shionogi: Grant/Research Support

